# PTH-dependence of the effectiveness of cinacalcet in hemodialysis patients with secondary hyperparathyroidism

**DOI:** 10.1038/srep19612

**Published:** 2016-04-13

**Authors:** Tadao Akizawa, Noriaki Kurita, Masahide Mizobuchi, Masafumi Fukagawa, Yoshihiro Onishi, Takuhiro Yamaguchi, Alan R. Ellis, Shingo Fukuma, M. Alan Brookhart, Takeshi Hasegawa, Kiyoshi Kurokawa, Shunichi Fukuhara

**Affiliations:** 1 Division of Nephrology, Department of Medicine, Showa University School of Medicine, Tokyo, Japan; 2 Department of Innovative Research and Education for Clinicians and Trainees (DiRECT), Fukushima Medical University Hospital, Fukushima, Japan; 3 Center for Innovative Research for Communities and Clinical Excellence (CIRC2LE), Fukushima Medical University, Fukushima, Japan; 4 Institute for Health Outcomes and Process Evaluation Research (iHope International), Kyoto, Japan; 5 Department of Healthcare Epidemiology, School of Public Health in the Graduate School of Medicine, Kyoto University, Kyoto, Japan; 6 Division of Nephrology, Endocrinology and Metabolism, Tokai University School of Medicine, Isehara, Japan; 7 Division of Biostatistics, Tohoku University Graduate School of Medicine, Sendai, Japan; 8 Cecil G. Sheps Center for Health Services Research, University of North Carolina at Chapel Hill, Tokyo, Japan; 9 Institute for Advancement of Clinical and Translational Science (iACT), Kyoto University Hospital, Kyoto, Japan; 10 Department of Epidemiology, Gillings School of Global Public Health, University of North Carolina at Chapel Hill, Tokyo, Japan; 11 Division of Nephrology, Department of Internal Medicine, Showa University Fujigaoka Hospital, Yokohama, Japan; 12 National Graduate Institute for Policy Studies, Tokyo, Japan

## Abstract

Cinacalcet lowers parathyroid hormone levels. Whether it can prolong survival of people with chronic kidney disease (CKD) complicated by secondary hyperparathyroidism (SHPT) remains controversial, in part because a recent randomized trial excluded patients with iPTH <300 pg/ml. We examined cinacalcet’s effects at different iPTH levels. This was a prospective case-cohort and cohort study involving 8229 patients with CKD stage 5D requiring maintenance hemodialysis who had SHPT. We studied relationships between cinacalcet initiation and important clinical outcomes. To avoid confounding by treatment selection, we used marginal structural models, adjusting for time-dependent confounders. Over a mean of 33 months, cinacalcet was more effective in patients with more severe SHPT. In patients with iPTH ≥500 pg/ml, the reduction in the risk of death from any cause was about 50% (Incidence Rate Ratio [IRR] = 0.49; 95% Confidence Interval [95% CI]: 0.29–0.82). For a composite of cardiovascular hospitalization and mortality, the association was not statistically significant, but the IRR was 0.67 (95% CI: 0.43–1.06). These findings indicate that decisions about using cinacalcet should take into account the severity of SHPT.

In people with advanced chronic kidney disease (CKD), secondary hyperparathyroidism (SHPT) can cause bone-related complications. Those complications include high levels of parathyroid hormone (PTH) and mineral abnormalities such as hypercalcemia and hyperphosphatemia. Correcting these abnormalities is important because they are associated with cardiovascular disease and mortality, which occurs relatively frequently in people with CKD^1^,^2^,^3^. Cinacalcet is a calcimimetic drug that inhibits the secrection of PTH and can reduce the risk of mineral abnormalities^4^,^5^. By retarding the progression of vascular calcification mediated by hypercalcemia and hyperphosphatemia^6^, cinacalcet might prevent death, and particularly death due to cardiovascular disease. However, results of previous studies of cinacalcet’s effect on cardiovascular disease and mortality were not definitive^7^, and may not be generalizable to many clinical settings.

In the Evaluation of Cinacalcet Therapy to Lower Cardiovascular Events (EVOLVE) study, cinacalcet’s efficacy was evaluated with a composite outcome of cardiovascular disease and all-cause death in adults with CKD stage 5D (dialysis). In that study, the difference between the cinacalcet and placebo groups was not statistically significant (hazard ratio 0.93)^8^. However, about 20% of the patients in the placebo group did not continue receiving placebo and instead began receiving cinacalcet. By design, the intention to treat (ITT) analysis in the randomized trial did not account for that crossover. In addition, a large percentage patients in the cinacalcet group discontinued the active drug, because of adverse effects. One consequence was that the power to detect cinacalcet’s effect in patients who were randomly assigned to receive it was probably reduced.

In addition, patients in that study might differ in important ways from patients in many clinical-practice settings. First, CKD stage 5D patients with SHPT are, on average, 7 to 8 years older than the participants in the EVOLVE study^9^,^10^. Second, to maintain intact PTH (iPTH) levels in the range of, for example, 150 to 300 pg/ml^11^, patients can be treated with cinacalcet even if their serum iPTH is <300 pg/ml, in which case they would not have been included in the EVOLVE study. Also, cinacalcet’s effect might depend on the serum iPTH level. Specifically, cinacalcet reduces the volume of the parathyroid glands and it reduces abnormally high levels of mineral and bone disorder (MBD) markers, but those effects are greater in patients with larger parathyroid glands and they are greater in patients with higher serum iPTH levels^12^,^13^. Therefore it is important to ask whether the effectiveness of cinacalcet against clinically important events is, similarly, greater in patients with higher serum iPTH levels. That is the question we sought to answer in the present study. The answer will allow physicians to inform their patients of the effects they can expect if they begin taking cinacalcet, including the different effects that might be expected given different severities of SHPT.

In Japan, cinacalcet has been commercially available since January 2008 and since then its use has gradually increased. With little or no prospect for enrolling patients in a randomized trial once cinacalcet was newly-authorized for clinical use, instead, just before cinacalcet became available, we started a large, prospective, observational study, the “Mineral and Bone Disorders Outcomes Study for Japanese CKD Stage 5D Patients” (MBD-5D). Using pre-specified protocols^14^, we analyzed data from the MBD-5D with marginal structural models (MSMs), to account for time-dependent confounding. Applying MSMs to these cinacalcet-naïve patients allowed us to better estimate the effects of cinacalcet initiation on death due to any cause and death due to cardiovascular disease in clinical practice. This is similar to ITT analysis, except that untreated patients are allowed to switch cinacalcet arms. This analytic approach of employing MSMs as a kind of ITT analyses has been used in other studies^15^.

## Results

In the subcohort (patients selected at ramdom from the whole cohort: see “sample sizes and study designs” section) the mean age was 61.9 years and the median dialysis vintage was 8.3 years (Table 1). At baseline (i.e. before cinacalcet was marketed), 1328 (40.5%) of the patients had iPTH values ≥300 pg/ml , and the prevalences of guideline-defined hypercalcemia (corrected serum calcium level >10 mg/dl) and hyperphosphatemia (serum phosphorus level >6.0 mg/dl)^16^ were 25% and 32%, respectively (Table 1). Cinacalcet was prescribed at any time during follow-up to 1384 (42%) of the subcohort patients (Table 2). Baseline characteristics of the cases (patients who experienced “death due to cardiovascular disease” or “death due to any cause”; see “Sample sizes and study designs” section) outside the subcohort are shown in Table S1. When compared with patients who had never received cinacalcet, those who did receive cinacalcet were younger, less likely to have diabetic nephropathy, and less likely to have cardiovascular disease (Table 2). As a group, their history of dialysis treatment was longer, and they were more likely to have high levels of calcium, phosphorus, and iPTH. They were more likely to have received an intravenous vitamin D receptor activator (VDRA), were less likely to have received a calcium-based phosphate binder, and overall they had higher values of Kt/V, albumin, and creatinine. Those in higher iPTH categories received intravenous VDRA more often, and they had more severe hypercalcemia and hyperphosphatemia (Table 1). Differences in characteristics among the patients with or without cinacalcet prescription by baseline iPTH categories were similar to those in the total subcohort (Table 2).

**Figure 1:**
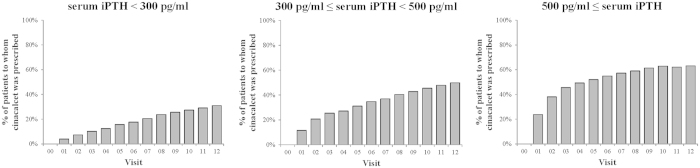
Changes in the proportion of patients receiving cinacalcet over the 3-year study period, stratified by baseline iPTH category . Changes in the proportion of patients receiving cinacalcet are shown for 3 categories of serum iPTH at baseline (n = 1,948 for <300 pg/ml, n = 824 for 300–<500 pg/ml, and n = 504 for ≥500 pg/ml). Visit 0 indicates the baseline (December 2007). The time between visits was 3 months. In January 2008 (within visit 1), cinacalcet was approved for use in clinical practice in Japan. Data were derived from the subcohort (n = 3,276). The numbers of patients analyzed gradually decreased to 2,469 at visit 12, due to death, loss to follow-up, and other reasons.

**Figure 2:**
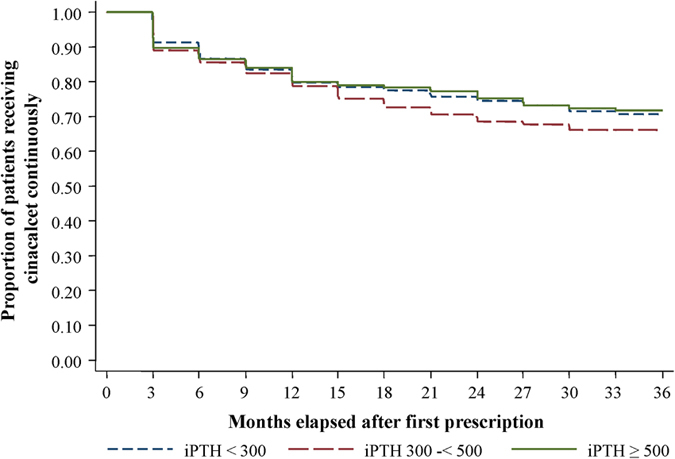
Proportion of patients receiving cinacalcet continuously over the study period, stratified by baseline iPTH category. The proportion of patients receiving cinacalcet continuously is shown for 3 categories of serum iPTH at baseline (n = 624 for <300 pg/ml, n = 420 for 300–<500 pg/ml, and n = 340 for ≥500 pg/ml). Those 3 groups did not differ (P = 0.23 by log-rank test). Three months after the first prescription was the first visit at which patients were considered to be receiving cinacalcet, because the time between visits was 3 months. Data were derived from the subcohort (n = 1,384). The number of patients analyzed gradually decreased, and it was 313 at the 36th month after the first prescription, due to the end of follow-up, death, loss to follow-up, and other reasons.

The proportion of patients receiving cinacalcet increased monotonically from 0% at visit 0 to 42% at visit 12 (Fig. S1, intervals between visits were 3 months). At visit 12, the median (interquartile range) dose of cinacalcet in the patients who received it was 25 mg/day (25 to 50 mg/day). The estimated proportion of patients receiving cinacalcet who continued to receive it for 36 months (i.e., 12 visits) decreased from the time of the first prescription but still it was 70% in the 36th month after the first prescription (Fig. S2). When patients were stratified by baseline iPTH categories, the proportions of patients receiving cinacalcet at visit 12 differed by more than 30 percentage points: 30.9%, 49.9%, and 63.2% in those with baseline iPTH levels <300 pg/ml, 300–<500 pg/ml, and more than 500 pg/ml, respectively (Fig. 1). However, the estimated proportions of patients who had *continuously* received cinacalcet for 36 months after the first prescription differed only very slightly: 70.7%, 66.1%, and 71.8% among the three baseline iPTH levels (log-rank P = 0.23) (Fig. 2).

A total of 1226 deaths occurred in the total cohort during the observation period, of which 521 (41.8% of all deaths) were due to cardiovascular disease (Table S2), and 159 (13.0% of all deaths) were of “unknown” cause and thus were not included among those due to cardiovascular causes. The crude rates of death due to any cause, death due to cardiovascular disease, and cardiovascular hospitalization or death were 5.49, 2.07, and 13.2 per 100 person-years, respectively.

The iPTH level at baseline modified the effect of cinacalcet’s initiation on clinical outcomes. In higher categories of baseline iPTH, the apparent benefits of cinacalcet were larger (Table 3). In particular, in patients with baseline iPTH levels ≥500 pg/ml, cinacalcet initiation was associated with lower incidence rate ratio (IRR) for death due to any cause (adjusted IRR 0.49, 95% CI 0.29–0.82). For cardiovascular hospitalization or death due to any cause, although the association was not statistically significant, the adjusted IRR was less than 1 (IRR = 0.67; 95% CI 0.43–1.06). In contrast, in patients with baseline iPTH levels <300 pg/ml, cinacalcet initiation was not associated with any of the 3 clinical outcomes. Graded magnitudes of cinacalcet’s effects across baseline iPTH categories were consistent among all 3 clinical outcomes.

When analyses were restricted to patients with iPTH levels ≥300 pg/ml (an inclusion criterion of the EVOLVE study), cinacalcet initiation was associated with a lower incidence of cardiovascular hospitalization or death due to any cause (adjusted IRR 0.71, 95% CI 0.53–0.94) (Table 4). For death due to any cause, although the association was not statistically significant, the adjusted IRR was less than 1 (IRR = 0.75; 95% CI 0.55–1.03). Cinacalcet’s association with cardiovascular death was not statistically significant (adjusted IRR 0.90, 95% CI 0.56–1.47).

In the sensitivity analyses of effect modifications in the total cohort and primary effects in patients with iPTH levels ≥300 pg/ml, the directions of associations between cinacalcet initiation and IRs of the 3 clinical outcomes, and the precisions of those estimates, were similar to the results of the main analyses (Tables S3, S4).

## Discussion

In this large prospective study of CKD stage 5D patients with SHPT receiving hemodialysis, cinacalcet initiation was more effective in patients who had higher levels of iPTH at baseline, on two important clinical outcomes: death due to any cause and a composite of cardiovascular hospitalization and mortality. Specifically, in patients with baseline iPTH levels ≥300 pg/ml cinacalcet initiation was associated with lower incidences of cardiovascular hospitalization or death due to any cause.

Several strengths of the present study warrant mention. First, the effects estimated by MSMs can be interpreted as average differences between two rates: the rate of outcomes that would be expected if all patients initiated cinacalcet and the rate that would be expected if none received it. By starting the study before cinacalcet was marketed, we acquired information regarding cinacalcet use from the time that it was first prescribed. Enrolling only new cinacalcet users who had pretreatment baseline measurements prevents survivorship bias. Analyzing those data together with variables likely to affect cinacalcet prescription (e.g., PTH, calcium, and VDRA), we adjusted for the effects of those time-dependent confounders. With MSMs, we adjusted for those confounders without removing their intermediate effect caused by previous cinacalcet use. Probably because of these advantages of MSMs, their use is becoming common in nephrology research^17^,^18^,^19^. Second, the results indicating iPTH-dependence of cinacalcet’s effects came from circumstances found in regular clinical practice. This point is important because SHPT treatment varies between dialysis facilities, and dialysis patients vary widely regarding comorbid conditions and severity of SHPT. Third, regarding generalizability, we note that in age, vintage, and comorbidities the patients in this study were similar to a randomly-selected sample of dialysis patients in Japan^20^.

Cinacalcet initiation was more effective in patients with more severe iPTH levels. The EVOLVE study found no statistically significant effect of cinacalcet on cardiovascular outcomes (hazard ratio 0.93, 95% CI 0.85–1.02), which appears to conflict with the results of the present study restricted to patients with baseline iPTH levels ≥300 pg/ml. However, interpretation of that hazard ratio is complicated by imbalance in prognostic factors at baseline, by discontinuation of active cinacalcet therapy among two thirds of patients in the cinacalcet group, and by 20% crossover from the placebo group^21^. In the present study, the analyses were designed to simulate ITT analyses except that untreated patients were allowed to switch to the cinacalcet arm, and their results might be less diluted by non-adherence to cinacalcet than were the results of the EVOLVE study, partly because the estimated proportion of patients receiving cinacalcet continuously was high during the 3 years after the first prescription. Consistent with this observation, compliance regarding drug continuation and dialysis treatment are better in Japan than in other countries^22^,^23^. In addition, we could also examine effects of cinacalcet’s initiation in patients with baseline iPTH levels <300 pg/ml, who would have been excluded from the EVOLVE study. Among them, cinacalcet was associated with none of the 3 clinical outcomes. Further study is warranted to determine whether patients with iPTH <300 pg/ml can benefit from cinacalcet. These findings may be strengthened by three facts: First, SHPT is treated to lower target values in Japan than in the US^16^. Second, in 41.2% of the dialysis facilities in this MBD-5D study (a representative random sample of dialysis facilities in Japan) cinacalcet can be started at iPTH levels of 150–300 pg/ml^24^. Third, the very high iPTH levels seen in African-Americans^25^ are less common in Japan.

Although the cohort size was large and the follow-up of 3 years offered a good time range to report on meaningful outcomes, the effects of cinacalcet’s initiation on cardiovascular death were not statistically significant, even in patients with high levels of serum iPTH. A number of explanations are possible. In particular, we note that 13% of deaths were of unknown cause. If any of those deaths were actually of cardiovascular cause, then they were misclassified, which might have led to results being not statistically significant. In addition, only 4.6% of deaths were attributed to sudden death in our study. The EVOLVE post-hoc study suggested that cinacalcet could benefit the cardiovascular system by attenuating nonatherosclerotic cardiovascular events like sudden death, which accounted for 24.5% of deaths in the EVOLVE trial^26^. Also, the ITT-like analysis cannot account for dilution of effects caused by discontinuation of cinacalcet. In addition, when we calculated the sample size we overestimated both the size of the effect of a drug such as cinacalcet and the numbers of clinical outcomes^14^. It is clear in retrospect that our study was underpowered, as both the event rate and the effect size were overestimated in the sample-size computations. Discontinuation of cincalcet by some patients also contributed to underpowering. This could explain the failure to differentiate cinacalcet’s effects on some clinical outcomes from the null.

As possible explanations for the finding that cinacalcet was more effective in patients with higher iPTH levels, we note that the magnitudes of the reductions in iPTH and phosphorus are greater in patients with higher levels of iPTH at baseline^13^. In addition, although median cinacalcet dose prescribed in our study population was 25 mg/day (interquartile range 25–50 mg/day), our previous study showed that initiating cinacalcet resulted in better iPTH control, with an absolute difference of 25%^27^. That previous study also showed that initiating cinacalcet and reducing VDRA dosage resulted in 9% and 12% increases in the number s of patients who were within the guideline-specified calcium and phosphorus ranges. Initiating cinacalcet alone while maintaining the VDRA dosage resulted in an 8% increase in the number of patients who were within the guideline-specified calcium range^27^.Thus patients with higher levels of MBD markers might particularly benefit from the reduction of MBD markers by initiation of cinacalcet with subsequent VDRA adjustment.

Several limitations of this study should be mentioned. First, we might not have measured all of the important influences on cinacalcet prescription. However, several sensitivity analyses with more covariates than the primary analyses gave results similar to those of the primary analyses, which indicates that the main findings are unlikely to have been influenced by residual confounding. Although cinacalcet users were younger, much more likely to have longer vintage, less comorbidity, and glomerulonephritis as the primary renal disease, those covariates were included in the MSMs. A major implication of our study is that differences in iPTH levels are causally related to differences in cinacalcet’s effectiveness with regard to important clinical outcomes. Imbalances in measured and unmeasured factors are unlikely to explain the variation in cinacalcet’s effectiveness related to iPTH levels.

The second limitation is that although several iPTH assay methods were available in Japan, information on the specific assay used for iPTH in each center could not be obtained. Third, multiple inferences were made but no adjustment was considered for the overall type-I error rate. Fourth, although the proportion of patients receiving cinacalcet continuously was described, we could not track actual cinacalcet use over time. However, compliance regarding drug continuation is better in Japan than in other countries^22^,^23^, and in Japanese dialysis practice the patient’s adherence is regularly determined every two weeks or every month, we believe it unlikely that the lack of tracking of cinacalcet prescriptions lead to misclassification. Fifth, we could not measure other MBD-related serum biomarkers such as bone specific alkaline phosphatase. Sixth, because patients could begin to receive cinacalcet at any time, the period between baseline iPTH measurement and cinacalcet initiation varied from 3–36 months, whereas in a randomized trial the baseline variables would be measured immediately prior to treatment initiation. However, our MSMs controlled for the variation in iPTH levels between baseline and cinacalcet initiation.

In conclusion, in this large unselected group of CKD stage 5D patients in Japan who had SHPT and received hemodialysis, cinacalcet was more beneficial in patients with higher iPTH levels on death from any cause and on a composite of cardiovascular hospitalization and mortality. These findings can inform the decisions of physicians and dialysis patients as they consider whether or not to use cinacalcet, given the severity of a patient’s SHPT.

## Methods

The MBD-5D was a 3-year prospective case-cohort and cohort study. Because this study was an observational study using anonymized data collected during routine practice, informed consent was not mandatory according to the ethical guidelines for epidemiological research in Japan^28^. The study protocol and the waiver of informed consent were approved by a central ethics committee at Kobe University’s School of Medicine (No. 754). The study was conducted in accordance with the Declaration of Helsinki and the ethical guidelines for epidemiological research in Japan^28^.

### Target population

The target population was CKD-5D patients with SHPT who received maintenance hemodialysis. Eligible patients were i) those receiving hemodialysis at one of the participating facilities as of 1 January 2008 and ii) either those with iPTH concentration ≥180 pg/mL or those who were receiving an intravenous Vitamin D receptor activator (VDRA) (calcitriol or maxacalcitol) or an oral active VDRA (falecalcitriol, the only oral VDRA approved in Japan for SHPT treatment). Patients who had been receiving dialysis for less than 3 months were excluded. From 86 facilities across Japan, 8229 patients were registered in the study cohort. Data were collected until January 2011.

### Outcomes and exposures

We examined three clinical outcomes: (1) death due to any cause, (2) death due to cardiovascular disease, and (3) single cardiovascular hospitalization or death due to any cause, whichever came first (Table S5). We examined the effects of “cinacalcet initiation” as the primary exposure. Specifically, before cinacalcet initiation all patients in the study were considered to be in the non-initiation arm. Once cinacalcet was initiated, all patients to whom it was prescribed were considered to be in the cinacalcet arm until the end of follow-up. As this was an observational study, the decision to prescribe or not prescribe cinacalcet was made by each physician. Prescription information was extracted from medical records, and was handled as a time-dependent variable. Misclassification due to primary non-adherence (not filling a prescription) is unlikely in this study, because patients in Japan usually go to a pharmacy soon after receiving a physician’s prescription, that is, about every two weeks or every month. In regular dialysis practice in Japan, at the time of each patient’s visit to a hemodialysis facility, a staff nurse verifys that the previously given prescription has been filled and the medication has been taken regularly.

### Sample sizes and study designs

**Figure 3:**
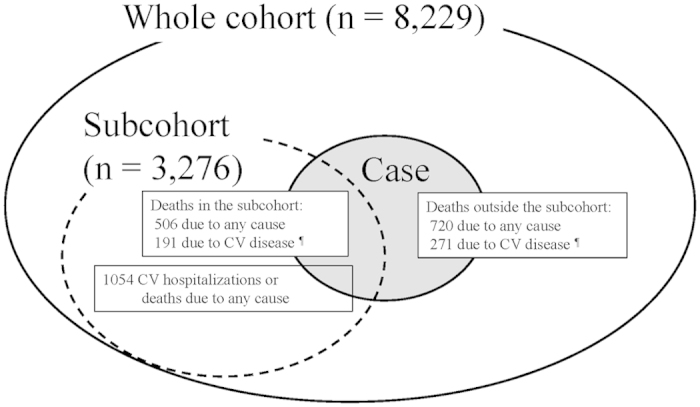
Study design of the MBD-5D. The study has a “whole cohort” (large solid circle) comprising all patients enrolled and a “subcohort” (dotted circle) comprising a randomly selected 40% of the whole cohort. From 86 facilities, all 8,229 dialysis patients with secondary hyperparathyroidism were registered, and 3,276 were selected into the subcohort. Data were collected prospectively from the subcohort, and retrospectively from those outside the subcohort who died. In total, there were 1,226 deaths due to any cause (small solid circle with gray color) and 462 deaths due to cardiovascular disease. As for death due to any cause, data from 3,996 patients (3,276 patients in the subcohort, among whom there were 506 deaths, together with 720 deaths among patients outside the subcohort) were analyzed as a case-cohort study. Similarly, as for death due to cardiovascular disease, data from 3,547 patients (3,276 patients in the subcohort, among whom there were 191 deaths, together with 271 deaths among patients outside the subcohort) were analyzed as a case-cohort study. As for cardiovascular hospitalization or death due to any cause, 1,054 cases were observed in the subcohort, and data from 3,276 subcohort patients were analyzed as a cohort study. CV: cardiovascular.

As the MBD-5D study was designed to answer more than one research question (associations between mineral abnormalities and outcomes, and associations between cinacalcet and outcomes), the sample size was not determined for specific treatments^14^. However, sample size was determined as follows: for death due to cardiovascular disease, (1) the expected rate was 2.5 deaths per 100 person-years during the 3-year follow up period, (2) the effect size of a drug was 20% to 25% of the relative risk, (3) the proportion of patients to whom the drug was prescribed was one-third. With those values, 6000–7500 patients would be required for 80% power with a two-sided alpha of 0.05. We did not have the financial resources needed to hire clinical research coordinators to collect data repeatedly from all of the patients (the “whole cohort”, n = 8229), and therefore we conducted a case-cohort study. Patients were randomly sampled from the whole cohort to yield a “subcohort”. Although data were prospectively and repeatedly collected only from patients in the subcohort, drug’s effect could be estimated by analyzing data from the whole cohort^14^,^29^. (Figure 3) Specifically, the case-cohort analysis included records of all patients in whom an outcome event occurred as well as all records of subcohort patients in whom an outcome event did not occur. The former received a weight of 1.0 and the latter received a weight determined by the inverse of the sampling fraction, to account for the fact that non-cases outside the subcohort were not observed^29^. The case-cohort design was used for “death due to cardiovascular disease” and for “death due to any cause” (cases) (Fig. 3). To examine whether a drug was associated with reduced cardiovascular hospitalization or death due to any cause in the subcohort, the sampling fraction used was 0.4 (i.e. the subcohort n = 3276) based on the following assumptions: (1) the expected rate of cardiovascular hospitalization or death due to any cause was assumed to be 13.0 events per 100 person-years during the 3-year follow-up period, (2) the effect size of a drug was assumed to be 20% to 25% of the relative risk, and (3) the proportion of patients to whom the drug was prescribed was one-third. With those values, 3000 patients would be required for 80% power with a two-sided alpha of 0.05^14^. A cohort design was used for cardiovascular hospitalization or death due to any cause. Details of the participants in each part of the study are given in Figure S3. As reported previously, the randomly-selected subcohort and the whole cohort were similar in terms of age, gender, and iPTH at baseline^10^.

### Covariates

Covariates used in the primary analyses included baseline (time of entry into cohort) patient characteristics (age, gender, vintage, primary renal disease, cardiovascular disease, lung disease, liver disease, malignancy, and history of parathyroidectomy), MBD-related serum markers (calcium, phosphorus, and iPTH), prescriptions for MBD-related drugs (VDRAs and phosphate binders), and other potential confounders (dialysate calcium concentration, Kt/V, albumin, BMI, and hemoglobin).

### Data collection

Data were collected from 86 dialysis facilities by trained staff. Data on demographics and comorbidities were collected at the time of enrollment (visit 0). Data on MBD markers and treatments were collected at the time of enrollment (visit 0) and every 3 months for 3 years (visits 1–12). Data were collected every 6 months for the other time-dependent variables, prospectively for the subcohort patients and retrospectively for cases outside the subcohort. The laboratory data used were those measured closest to the end of each visit. Serum iPTH levels (reference 10–65 pg/ml) were measured in 73 facilities. Serum whole PTH levels measured by a third-generation PTH assay (immunoradiometric assay; reference 9–39 pg/ml) were used in 13 facilities and converted to iPTH levels: iPTH = whole PTH × 1.7^16^. Serum calcium levels were corrected for albumin concentration using the modified Payne method, which is commonly used in Japanese dialysis settings^16^.

### Statistical analysis

Crude mortality rate and cardiovascular mortality rate were estimated using all cases and the subcohort patients. Crude incidence rates (IRs) for cardiovascular hospitalization and death due to any cause were estimated from the subcohort data. The crude rates were calculated for those 3 clinical outcomes, stratified by the status of cinacalcet use.

### Marginal structural models

Marginal structural models (MSMs) were used to estimate cinacalcet’s effects on clinical outcomes^15^,^30^,^31^. MSMs appropriately account for effects of potential time-dependent confounders such as iPTH and calcium, which are also a potential source of confounding by indication^30^. They are affected by previous cinacalcet use and they are also predictors of future cinacalcet use and of clinical outcomes^32^. In addition, MSMs allow us to compare two IRs: the IR that would be expected if all study patients were treated and the IR that would be expected if none of the study patients were treated. IRR of clinical outcomes were estimated for cinacalcet initiation in comparison with non-initiation.

For each 3-month period ending at visit *t*, the incidence of each clinical outcome was modeled as a function of whether cinacalcet had been initiated at or before the previous 3-month period (visit *t-1*). Weights were calculated from the inverse of the probability of the cinacalcet-prescription history a patient actually had during each 3-month period of follow-up until cinacalcet initiation. These probabilities were predicted from a pooled logistic regression model. In that model, the dependent variable was cinacalcet prescription at a given visit *t-1*. The independent variables were time-dependent confounders at visit *t-2* (calcium, phosphorus, iPTH, VDRA, phosphate binder, several product terms of these MBD biomarkers and treatments, dialysate calcium, Kt/V, albumin, BMI, hemoglobin), baseline covariates (age, gender, vintage, primary renal disease, cardiovascular disease, lung disease, liver disease, malignancy, history of parathyroidectomy), and visit number. Similarly, censoring weighting was calculated to account for loss to follow-up (n = 143), switch to peritoneal dialysis (n = 2) or renal transplantation (n = 5).

Weighted Poisson regression with robust variance was used to estimate the IRRs. Any data missing at baseline were replaced by their mean or median values, or by predicted values from linear regression models. Missing data were imputed by carrying the last observation forward. We used these simple imputation methods for two reasons: First, there were very few missing values of MBD-related markers of interest (<0.2% at baseline, and about 2% during the follow-up, except for 6% in iPTH). Second, for laboratory values, the last-observation-forward technique makes sense in that physicians would likely use a similar method to make prescription decisions.

To estimate modification of effect of cinacalcet intiation by iPTH at baseline, we used three categories of baseline iPTH: <300 pg/ml, 300–<500 pg/ml, and 500 ≥ pg/ml. The cut-off value of 300 pg/ml was based on the upper limit of the targeted range in the KDOQI guideline and on the inclusion criteria in the EVOLVE study^8^,^11^. The cut-off value of 500 pg/ml was based on the reference for parathyroid intervention therapy recommended in the Japanese guidelines, as patients with iPTH ≥500 pg/ml are considered to have a higher likelihood of having nodular hyperplasia in the parathyroid glands^14^. These three categories were entered into the weighted Poisson regression model with interaction terms with cinacalcet initiation^32^.

To estimate cinacalcet’s effects in patients similar to those in the EVOLVE study, further analyses were done using data only from those patients with an iPTH level ≥300 pg/ml (the “restricted” analyses).

Sensitivity analysis of effect modification was done, with baseline iPTH categories included in models estimating treatment weights or with more covariates (covariates in the primary analysis, plus dementia, other central nervous disease, baseline creatinine and total cholesterol, time-varying values of total iron, ferritin, and CRP, and specific cardiovascular conditions: coronary artery disease, atrial fibrillation, arrhythmia, congestive heart failure, cerebrovascular disease, peripheral vascular disease, aortic disease, pacemaker, or other cardiovascular conditions. About 20% of the data on total iron, ferritin, and CRP were missing, so the function aRegImpute in R was used to make an imputed dataset^33^.). Sensitivity analyses were also done with or without more covariates for the patients restricted to those with baseline iPTH levels ≥300 pg/ml (an inclusion criterion of the EVOLVE study).

P values < 0.05 were taken as indicators of statistical significance. Using SAS 9.2 (SAS Institute, Cary, NC), N.K. and Y.O. analyzed the data first. Then A.E.R. used his original SAS coding and obtained the same results.

## Additional Information

**How to cite this article**: Akizawa, T. *et al.* PTH-dependence of the effectiveness of cinacalcet in hemodialysis patients with secondary hyperparathyroidism. *Sci. Rep.* **6**, 19612; doi: 10.1038/srep19612 (2016).

## Supplementary Material

Supplementary Information

Supplementary Information

## Figures and Tables

**Table 1:** Baseline characteristics of Subcohort Patients, including stratification by iPTH category^a^.

Characteristics (%)	Baseline Serum iPTH, pg/ml			Total^b^
	<300	300–<500	≥500	
	n = 1,948	n = 824	n = 504	n = 3,276
Demographics				
Age, yr	62.7 (12.8)	61.5 (12.8)	59.7 (11.9)	61.9 (12.7)
Gender				
Women	37%	41%	39%	38%
Men	63%	59%	61%	62%
Renal disease				
Glomerulonephritis	41%	45%	58%	45%
Diabetic nephropathy	28%	21%	13%	24%
Other diseases	30%	34%	29%	31%
Vintage, yr	6.9 (1.5, 20.7)	8.9 (1.7, 21.7)	11.6 (3.1, 24.9)	8.3 (1.6, 22.1)
Body mass index, kg/m^2^	21.4 (3.4)	21.5 (3.8)	21.2 (3.3)	21.4 (3.5)
Comorbid conditions				
Cardiovascular conditions	61%	61%	57%	60%
Coronary artery disease	26%	24%	24%	25%
Atrial fibrillation	7%	7%	7%	7%
Other arrhythmia	12%	13%	12%	12%
Congestive heart failure	9%	7%	6%	8%
Cerebrovascular disease	10%	12%	11%	11%
Peripheral vascular disease	19%	18%	17%	19%
Aortic disease	7%	6%	5%	7%
Others	14%	14%	15%	14%
Diabetes mellitus	36%	29%	18%	31%
Lung disease	7%	6%	9%	7%
Liver disease	14%	14%	15%	14%
Malignancy	4%	6%	6%	5%
History of parathyroidectomy	5%	6%	10%	6%
Dementia	3%	4%	3%	3%
Other central nervous disease	8%	9%	10%	9%
Laboratory measurements and treatment variables				
Serum calcium^b^, mg/dl	9.4 (0.9)	9.4 (0.9)	9.7 (0.9)	9.5 (0.9)
<8.4 mg/dl	10%	10%	8%	10%
≥8.4–≤ 10.0 mg/dl	67%	64%	58%	65%
>10.0 mg/dl	23%	26%	34%	25%
Serum phosphorus, mg/dl	5.3 (1.3)	5.8 (1.4)	6.1 (1.5)	5.5 (1.4)
<3.5 mg/dl	6%	4%	2%	5%
≥3.5–≤ 6.0 mg/dl	69%	58%	48%	63%
>6.0 mg/dl	25%	38%	50%	32%
Serum iPTH, pg/ml	206 (91, 278)	371 (312, 464)	692 (524, 1183)	265 (124, 611)
VDRA				
Intravenous	46%	45%	66%	49%
Oral	34%	27%	12%	29%
None	20%	28%	22%	23%
Phosphate binder				
Both	23%	23%	24%	23%
Calcium-based	48%	42%	30%	44%
Not calcium-based	14%	20%	34%	18%
None	15%	15%	13%	15%
Dialysate calcium				
<3 mg/dl	52%	51%	52%	52%
≥3 mg/dl	48%	49%	48%	48%
Kt/V	1.41 (0.3)	1.42 (0.3)	1.45 (0.3)	1.42 (0.3)
Hemoglobin, g/dl	10.5 (1.2)	10.5 (1.1)	10.5 (1.2)	10.5 (1.2)
Serum albumin, g/dl	3.74 (0.4)	3.77 (0.4)	3.78 (0.3)	3.75 (0.4)
Serum creatinine, mg/dl	11.0 (2.9)	11.3 (2.9)	11.8 (2.8)	11.2 (2.9)
Serum cholesterol, mg/dl	155 (36)	154 (34)	154 (33)	154 (35)
Serum iron, μg/dl	59 (33, 95)	57 (32, 93)	58 (31, 92)	58 (33, 94)
Serum ferritin, ng/dl	124 (22, 394)	119 (18, 405)	91 (18, 316)	118 (20, 391)
Serum CRP, mg/L	1.1 (0.3, 10.2)	1.1 (0.3, 11)	1.0 (0.3, 8.4)	1.1 (0.3, 10)

^a^Mean (SD) are shown for normally distributed data; otherwise, median (p10, p90) are shown for non-normally distributed data.

^b^Corrected for albumin concentration using modified Payne method.

**Table 2:** Baseline characteristics of subcohort patients by cinacalcet use, including stratification by baseline iPTH category^a^.

Characteristics (%)	Total (n = 3,276)							
	By baseline iPTH, pg/ml							
	<300		300–<500		≥500		Total (n = 3,276)	
	Never(n = 1,324)	Ever(n = 624)	Never (n = 404)	Ever(n = 420)	Never (n = 164)	Ever(n = 340)			Never(n = 1,892)	Ever(n = 1,384)
Demographics								
Age, yr	64.4 (12.8)	59.1 (11.9)	64.0 (13.3)	59.1 (11.9)	62.6 (12.4)	58.3 (11.4)	64.1 (12.9)	58.9 (11.8)
Gender								
Women	37%	39%	37%	44%	42%	38%	37%	40%
Men	63%	61%	63%	56%	58%	62%	63%	60%
Renal disease								
Glomerulonephritis	37%	50%	37%	53%	57%	59%	39%	53%
Diabetic nephropathy	33%	19%	26%	16%	16%	11%	30%	16%
Other diseases	30%	31%	37%	31%	27%	30%	31%	31%
Vintage, yr	5.3 (1.2, 19.8)	9.7 (2.9, 22.4)	5.9 (0.9, 18.5)	11.4 (3.7, 23.5)	9.5 (1.5, 23.8)	12.7 (4.5, 25.5)	5.8 (1.1, 20.0)	11.0 (3.5, 24.0)
Body mass index, kg/m^2^	21.3 (3.6)	21.5 (3.1)	21.3 (4.0)	21.6 (3.5)	20.9 (3.1)	21.4 (3.4)	21.3 (3.6)	21.5 (3.3)
Comorbid conditions								
Cardiovascular	63%	57%	60%	61%	61%	54%	62%	57%
Coronary artery disease	27%	23%	25%	23%	29%	21%	27%	22%
Atrial fibrillation	7%	5%	6%	8%	11%	5%	7%	6%
Other arrhythmia	13%	12%	11%	15%	13%	11%	12%	13%
Congestive heart failure	9%	8%	10%	5%	7%	5%	9%	6%
Cerebrovascular disease	12%	7%	14%	10%	12%	10%	12%	9%
Peripheral vascular disease	20%	17%	19%	17%	20%	16%	20%	17%
Aortic disease	8%	6%	7%	6%	5%	5%	7%	6%
Others	15%	13%	14%	14%	13%	15%	15%	14%
Diabetes mellitus	41%	25%	36%	22%	26%	14%	39%	21%
Lung disease	8%	6%	7%	5%	9%	9%	8%	6%
Liver disease	14%	12%	15%	13%	15%	16%	15%	13%
Malignancy	5%	3%	8%	5%	8%	5%	6%	4%
History of parathyroidectomy	4%	6%	3%	10%	10%	10%	4%	8%
Dementia	4%	2%	5%	2%	3%	2%	4%	2%
Other central nervous disease	8%	8%	9%	10%	10%	10%	8%	9%
Laboratory measurements and treatment variables								
Serum calcium^b^, mg/dl	9.2 (0.8)	10.0 (0.7)	9.0 (0.8)	9.8 (0.7)	9.3 (1.0)	9.8 (0.8)	9.1 (0.8)	9.9 (0.7)
<8.4 mg/dl	15%	2%	19%	2%	17%	3%	15%	2%
≥8.4–≤ 10.0 mg/dl	73%	54%	70%	57%	62%	56%	72%	55%
>10.0 mg/dl	12%	44%	11%	41%	21%	41%	13%	43%
Serum phosphorus, mg/dl	5.2 (1.3)	5.5 (1.2)	5.6 (1.4)	5.9 (1.3)	6.0 (1.6)	6.2 (1.5)	5.4 (1.4)	5.8 (1.3)
<3.5 mg/dl	7%	4%	5%	2%	2%	1%	6%	3%
≥ 3.5–≤ 6.0 mg/dl	71%	66%	60%	57%	49%	48%	67%	59%
>6.0 mg/dl	22%	30%	35%	41%	49%	51%	27%	38%
Serum iPTH, pg/ml	202 (81, 273)	218 (118, 285)	362 (310, 461)	383 (317, 471)	681 (517, 1290)	696 (526, 1149)	236 (100, 473)	328 (168, 744)
VDRA								
Intravenous	40%	58%	29%	61%	54%	72%	39%	63%
Oral	36%	28%	37%	17%	22%	7%	35%	20%
None	24%	13%	34%	21%	24%	21%	26%	18%
Phosphate binder								
Both	19%	33%	20%	26%	23%	25%	19%	29%
Calcium-based	53%	37%	51%	33%	33%	28%	51%	33%
Not calcium-based	10%	22%	8%	32%	24%	38%	11%	29%
None	18%	8%	21%	9%	20%	9%	19%	9%
Dialysate calcium								
<3.0 mg/dl	54%	50%	53%	48%	54%	51%	54%	50%
≥3.0 mg/dl	46%	50%	47%	52%	46%	49%	46%	50%
Kt/V	1.38 (0.3)	1.46 (0.3)	1.38 (0.3)	1.46 (0.3)	1.47 (0.3)	1.44 (0.3)	1.39 (0.3)	1.46 (0.3)
Hemoglobin, g/dl	10.4 (1.2)	10.7 (1.2)	10.5 (1.2)	10.5 (1.1)	10.5 (1.3)	10.5 (1.1)	10.4 (1.2)	10.6 (1.1)
Serum albumin, g/dl	3.71 (0.4)	3.79 (0.4)	3.73 (0.4)	3.80 (0.3)	3.72 (0.4)	3.80 (0.3)	3.72 (0.4)	3.80 (0.3)
Serum creatinine, mg/dl	10.6 (2.9)	12.0 (2.7)	10.7 (2.9)	11.8 (2.7)	11.0 (2.7)	12.2 (2.8)	10.7 (2.9)	12.0 (2.7)
Serum cholesterol, mg/dl	154 (36)	155 (36)	157 (34)	151 (33)	156 (35)	152 (33)	155 (36)	153 (34)
Serum iron, μg/dl	58 (33, 96)	60 (33, 93)	56 (32, 94)	58 (34, 93)	53 (29, 90)	60 (33, 93)	57 (32, 95)	59 (33, 93)
Serum ferritin, ng/dl	126 (23, 412)	122 (20, 354)	123 (18, 436)	117 (18, 397)	83 (14, 308)	99 (18, 319)	121 (21, 408)	114 (19, 361)
Serum CRP, mg/L	1.1 (0.3, 11)	1.0 (0.3, 8.5)	1.1 (0.3, 13)	1.1 (0.4, 8.4)	1.5 (0.4, 8.0)	1.0 (0.3, 8.6)	1.1 (0.3 11)	1.0 (0.3, 8.5)

^a^Mean (SD) are shown for noikrmally distributed data; otherwise, median (p10, p90) are shown for non-normally distributed data.

^b^Corrected for albumin concentration using modified Payne method.

**Table 3:** Adjusted associations between cinacalcet use and clinical outcomes, computed using marginal structural models, stratified by baseline iPTH category^a^.

Baseline iPTH pg/ml	Adjusted RR	95% CI	p-value
Death due to any cause^b^			
<300	1.07	0.77–1.48	0.682
300–<500	0.88	0.61–1.29	0.517
≥500	0.49	0.29–0.82	0.007
Death due to cardiovascular disease^b^			
<300	0.92	0.56–1.50	0.725
300–<500	0.87	0.45–1.70	0.691
≥500	0.69	0.37–1.32	0.264
Cardiovascular hospitalization or death^c^			
<300	1.05	0.77–1.42	0.766
300–<500	0.71	0.47–1.05	0.087
≥500	0.67	0.43–1.06	0.087

RR: incidence rate ratio, 95% CI: 95% confidence interval.

^a^Estimated from weighted Poisson regression models. To calculate weight, probability of initiating cinacalcet was predicted by age, sex, vintage, primary renal disease, cardiovascular disease, lung disease, liver disease, malignancy, parathyroidectomy, time-varying value of VDRA, phosphate binder, serum Ca, serum inorganic Phosphorus, serum iPTH, dialysate Ca, Kt/V, serum Alb, BMI, Hgb, interaction terms of treatment variables and MBD variables, and visit number. To examine effect modification by baseline iPTH, baseline iPTH and its interaction with cinacalcet use were added to the weighted Poisson regression models.

^b^Estimated from case-cohort studies.

^c^Estimated from cohort study.

**Table 4:** Adjusted associations between cinacalcet use and clinical outcomes in patients with baseline iPTH ≥300 pg/ml, computed using marginal structural models^a^.

	*Adjusted RR*	*95% CI*	*p-value*
Death due to any cause^b^	0.75	0.55–1.03	0.073
Death due to cardiovascular disease^b^	0.90	0.56–1.47	0.683
Cardiovascular hospitalization or death^c^	0.71	0.53–0.94	0.016

RR: incidence rate ratio, 95% CI: 95% confidence interval.

^a^Estimated from weighted Poisson regression models. To calculate weight, probability of initiating cinacalcet was predicted by age, sex, vintage, primary renal disease, cardiovascular disease, lung disease, liver disease, malignancy, parathyroidectomy, time-varying value of VDRA, phosphate binder, serum Ca, serum inorganic Phosphorus, serum iPTH, dialysate Ca, Kt/V, serum Alb, BMI, Hgb, interaction terms of treatment variables and MBD variables, and visit number.

^b^Estimated from case-cohort studies.

^c^Estimated from cohort study.
