# Supplementary material for: PTH-dependence of the effectiveness of cinacalcet in hemodialysis patients with secondary hyperparathyroidism
Source: Sci Rep. 2016 Apr 13;6:19612. doi: 10.1038/srep19612 (PMC4829837; doi:10.1038/srep19612)
Supplement: Supplementary Information [file srep19612-s1.doc]

PTH-dependence of the effectiveness of cinacalcet in hemodialysis patients with secondary hyperparathyroidism

Tadao Akizawa, Noriaki Kurita, Masahide Mizobuchi, Masafumi Fukagawa, Yoshihiro Onishi, Takuhiro Yamaguchi, Alan R. Ellis, Shingo Fukuma, M. Alan Brookhart, Takeshi Hasegawa, Kiyoshi Kurokawa, and Shunichi Fukuhara

Supplementary Online Content

[Table S1. Baseline characteristics of Subcohort and Case Patients 2](#__RefHeading___Toc437260896)

[Table S2. Causes of death and hospitalization 4](#__RefHeading___Toc437260897)

[Table S3. Sensitivity analysis for associations between cinacalcet use and clinical outcomes using the marginal structural models, stratified by baseline iPTH categories^a^ 5](#__RefHeading___Toc437260898)

[Table S4. Sensitivity analysis for associations between cinacalcet use and clinical outcomes among patients with baseline iPTH ≥ 300 pg/ml using the marginal structural models^a^ 6](#__RefHeading___Toc437260899)

[Table S5. Outcomes ascertainment and adjudication 7](#__RefHeading___Toc437260900)

[Figure S1. Change in the proportion of patients receiving cinacalcet over the study period of 3 years 8](#__RefHeading___Toc437260901)

[Figure S2. Proportion of patients receiving cinacalcet continuously over the study period 9](#__RefHeading___Toc437260902)

[Figure S3. Case-cohort studies and cohort study design 10](#__RefHeading___Toc437260903)

[Appendix 11](#__RefHeading___Toc437260904)

# Table S1. Baseline characteristics of Subcohort and Case Patients

| | Characteristics (%) | | | | Subcohort^a^ |  | Cases outside subcohort | | | --- | --- | --- | --- | --- | --- | --- | --- | | Total^b^ |  | Death due to any cause | Death due to cardiovascular disease | | (n = 3,276) |  | (n = 720) | (n = 271) | | **Demographics** | | | |  |  |  |  | | **Age, yr** | | | | 61.9 (12.7) |  | 69.9 (10.8) | 68.7 (11.1) | | **Gender** | | *Men* | | 38%  62% |  | 36%  64% | 39%  61% | |  | | *Women* | | | **Renal disease**  *Glomerulonephritis*  *Diabetic nephropathy*  *Other diseases* | | | | 45%  24%  31% |  | 34%  37%  29% | 33%  39%  28% | | **Vintage, yr** | | | | 8.3 (1.6, 22.1) |  | 7.9 (1.8, 22.0) | 8.0 (1.8, 18.5) | | **Body mass index, kg/m^2^** | | | | 21.4 (3.5) |  | 20.4 (3.4) | 20.4 (3.2) | | **Comorbid conditions** | | | |  |  |  |  | | **Cardiovascular conditions** | | | |  | 60% | 77% | 78% | | Coronary artery disease | | | | 25% |  | 36% | 40% | | Atrial fibrillation | | | | 7% |  | 13% | 13% | | Other arrhythmia | | | | 12% |  | 14% | 14% | | Congestive heart failure | | | | 8% |  | 17% | 21% | | Cerebrovascular disease | | | | 11% |  | 17% | 18% | | Peripheral vascular disease | | | | 19% |  | 23% | 22% | | Aortic disease | | | | 7% |  | 10% | 13% | | Others | | | | 14% |  | 21% | 22% | | **Diabetes mellitus** | | | | 31% |  | 45% | 47% | | **Lung disease** | | | | 7% |  | 14% | 13% | | **Liver disease** | | | | 14% |  | 15% | 13% | | **Malignancy** | | | | 5% |  | 9% | 5% | | **History of parathyroidectomy** | | | | 6% |  | 2% | 1% | | **Dementia** | | | | 3% |  | 8% | 6% | | **Other central nervous disease** | | | | 9% |  | 8% | 8% | | **Laboratory measurements and treatment variables** | | | | | | | | | **Serum calcium^c^, mg/dl** | | | | 9.5 (0.9) |  | 9.4 (1.0) | 9.5 (0.9) | | *<8.4 mg/dl* | | | | 10% |  | 12% | 10% | | *≥ 8.4 – ≤ 10.0 mg/dl* | | | | 65% |  | 62% | 63% | | *> 10.0 mg/dl* | | | | 25% |  | 26% | 27% | | **Serum phosphorus, mg/dl** | | | | 5.5 (1.4) |  | 5.4 (1.4) | 5.5 (1.4) | | *<3.5 mg/dl* | | | | 5% |  | 6% | 6% | | *≥ 3.5 – ≤ 6.0 mg/dl* | | | | 63% |  | 65% | 60% | | *> 6.0 mg/dl* | | | | 32% |  | 29% | 34% | | **Serum iPTH, pg/ml** | | | | 265 (124, 611) |  | 273 (130, 640) | 272 (134, 648) | | **VDRA** | *Intravenous* | | | 49%  29%  23% |  | 44%  25%  31% | 45%  28%  27% | |  | *Oral* | | | |  | *None* | | | | **Phosphate binder** | *Both* | | | 23%  44%  18%  15% |  | 14%  41%  16%  29% | 12%  44%  17%  27% | |  | *Calcium-based* | | | |  | *Not calcium-based* | | | |  | *None* | | | | **Dialysate calcium** | | | *< 3.0 mg/dl* | 52%  48% |  | 53%  47% | 55%  45% | |  | | | *≥ 3.0 mg/dl* | | **Kt/V** | | | | 1.42 (0.3) |  | 1.41 (0.3) | 1.42 (0.3) | | **Hemoglobin, g/dl** | | | | 10.5 (1.2) |  | 10.2 (1.3) | 10.3 (1.3) | | **Serum albumin, g/dl** | | | | 3.75 (0.4) |  | 3.54 (0.4) | 3.59 (0.4) | | **Serum creatinine, mg/dl** | | | | 11.2 (2.9) |  | 9.5 (2.7) | 9.7 (2.6) | | **Serum cholesterol, mg/dl** | | | | 154 (35) |  | 152 (36) | 154 (37) | | **Serum iron, μg/dl** | | | | 58 (33, 94) |  | 55 (31, 93) | 55 (31, 91) | | **Serum ferritin, ng/dl** | | | | 118 (20, 391) |  | 121 (21, 407) | 123 (20, 416) | | **Serum CRP, mg/L** | | | | 1.1 (0.3, 10) |  | 2.2 (0.5, 20) | 2.4 (0.5, 20) |   ^a^Including cases within the subcohort.  ^b^Mean (SD) are presented for normally distributed data; otherwise, median (p10, p90) are presented for non-normally distributed data.  ^c^Corrected for albumin concentration using modified Payne method. |
| --- | --- | --- | --- | --- | --- | --- | --- | --- | --- | --- | --- | --- | --- | --- | --- | --- | --- | --- | --- | --- | --- | --- | --- | --- | --- | --- | --- | --- | --- | --- | --- | --- | --- | --- | --- | --- | --- | --- | --- | --- | --- | --- | --- | --- | --- | --- | --- | --- | --- | --- | --- | --- | --- | --- | --- | --- | --- | --- | --- | --- | --- | --- | --- | --- | --- | --- | --- | --- | --- | --- | --- | --- | --- | --- | --- | --- | --- | --- | --- | --- | --- | --- | --- | --- | --- | --- | --- | --- | --- | --- | --- | --- | --- | --- | --- | --- | --- | --- | --- | --- | --- | --- | --- | --- | --- | --- | --- | --- | --- | --- | --- | --- | --- | --- | --- | --- | --- | --- | --- | --- | --- | --- | --- | --- | --- | --- | --- | --- | --- | --- | --- | --- | --- | --- | --- | --- | --- | --- | --- | --- | --- | --- | --- | --- | --- | --- | --- | --- | --- | --- | --- | --- | --- | --- | --- | --- | --- | --- | --- | --- | --- | --- | --- | --- | --- | --- | --- | --- | --- | --- | --- | --- | --- | --- | --- | --- | --- | --- | --- | --- | --- | --- | --- | --- | --- | --- | --- | --- | --- | --- | --- | --- | --- | --- | --- | --- | --- | --- | --- | --- | --- | --- | --- | --- | --- | --- | --- | --- | --- | --- | --- | --- | --- | --- | --- | --- | --- | --- | --- | --- | --- | --- | --- | --- | --- | --- | --- | --- | --- | --- | --- | --- | --- | --- | --- | --- | --- | --- | --- | --- | --- | --- | --- | --- | --- | --- | --- | --- | --- | --- | --- | --- | --- | --- | --- | --- | --- | --- | --- | --- | --- | --- | --- | --- | --- | --- | --- | --- | --- | --- | --- | --- | --- | --- | --- | --- | --- | --- | --- | --- | --- | --- | --- | --- | --- | --- | --- | --- | --- | --- | --- | --- | --- | --- | --- | --- | --- | --- | --- | --- | --- | --- | --- | --- | --- | --- | --- | --- | --- | --- | --- | --- | --- | --- | --- | --- | --- | --- | --- | --- | --- | --- | --- | --- | --- | --- | --- | --- | --- | --- | --- | --- | --- | --- | --- | --- | --- | --- | --- | --- | --- | --- | --- | --- | --- | --- | --- | --- | --- | --- | --- | --- | --- | --- | --- | --- | --- | --- | --- | --- | --- | --- | --- | --- | --- | --- | --- | --- | --- | --- | --- | --- | --- | --- | --- | --- | --- | --- | --- | --- | --- | --- | --- | --- | --- | --- | --- | --- | --- | --- | --- | --- | --- | --- | --- | --- |

# Table S2. Causes of death and hospitalization

| | Death^a^ | | |  | Hospitalization^b^ | | | | --- | --- | --- | --- | --- | --- | --- | | Causes | Events (n) | Proportion |  | Causes | Events (n) | Proportion | | **Cardiovascular death** | 521 | 41.8% |  | **Cardiovascular hospitalization** | 1688 | 23.1% | | Sudden death | 56 | 4.6% |  | Myocardial infarction | 74 | 1.0% | | Myocardial infarction | 62 | 5.1% |  | Angina | 308 | 4.2% | | Heart failure | 100 | 8.2% |  | Silent myocardial ischemia | 12 | 0.2% | | Arrhythmia | 21 | 1.7% |  | Heart failure | 218 | 3.0% | | Cerebrovascular disorder | 110 | 9.0% |  | Arrhythmia | 92 | 1.3% | | Aortic disease | 8 | 0.7% |  | Cerebrovascular disease | 288 | 3.9% | | Others | 155 | 12.6% |  | Peripheral vascular disease | 192 | 2.6% | |  |  |  |  | Others | 504 | 6.9% | | **Noncardiovascular death** | 714 | 58.2% |  | **Noncardiovascular hospitalization** | 5627 | 76.9% | | Infectious disease | 149 | 12.2% |  | Infectious disease | 428 | 5.9% | | Malignant neoplasm | 109 | 8.9% |  | Malignant neoplasm | 308 | 4.2% | | Others | 297 | 24.2% |  | Vascular access trouble | 938 | 12.8% | | Unknown^c^ | 159 | 13.0% |  | Others | 3903 | 53.4% | |  |  |  |  | Unknown^c^ | 50 | 0.7% | | **Total** | 1,226 | 100% |  | **Total** | 7,315 | 100% |   ^a^Collected from the total cohort (n = 8,228).  ^b^Collected from the subcohort (n = 3,276).  ^c^Events of unknown cause were classified as noncardiovascular events. |
| --- | --- | --- | --- | --- | --- | --- | --- | --- | --- | --- | --- | --- | --- | --- | --- | --- | --- | --- | --- | --- | --- | --- | --- | --- | --- | --- | --- | --- | --- | --- | --- | --- | --- | --- | --- | --- | --- | --- | --- | --- | --- | --- | --- | --- | --- | --- | --- | --- | --- | --- | --- | --- | --- | --- | --- | --- | --- | --- | --- | --- | --- | --- | --- | --- | --- | --- | --- | --- | --- | --- | --- | --- | --- | --- | --- | --- | --- | --- | --- | --- | --- | --- | --- | --- | --- | --- | --- | --- | --- | --- | --- | --- | --- | --- | --- | --- | --- | --- | --- | --- | --- | --- | --- | --- | --- | --- | --- | --- | --- | --- | --- | --- | --- | --- | --- | --- | --- | --- | --- | --- | --- | --- | --- | --- | --- | --- |

# Table S3. Sensitivity analysis for associations between cinacalcet use and clinical outcomes using the marginal structural models, stratified by baseline iPTH categories^a^

|  | Baseline  iPTH pg/ml | Model 1 | | |  | Model 2 | | |
| --- | --- | --- | --- | --- | --- | --- | --- | --- |
|  |  | Adjusted  RR | 95% CI | p-value |  | Adjusted  RR | 95% CI | p-value |
| **Death due to any cause^b^** | < 300 | 1.07 | 0.77 – 1.47 | 0.702 |  | 1.14 | 0.83 - 1.58 | 0.408 |
|  | 300 - < 500 | 0.90 | 0.63 – 1.30 | 0.573 |  | 0.79 | 0.53 - 1.17 | 0.237 |
|  | ≥ 500 | 0.55 | 0.35 – 0.87 | 0.010 |  | 0.50 | 0.30 - 0.85 | 0.010 |
| **Death due to cardiovascular disease^b^** | < 300 | 0.88 | 0.54 – 1.43 | 0.604 |  | 0.99 | 0.59 – 1.67 | 0.973 |
|  | 300 - < 500 | 0.89 | 0.47 – 1.70 | 0.730 |  | 0.85 | 0.42 – 1.72 | 0.645 |
|  | ≥ 500 | 0.66 | 0.36 – 1.22 | 0.182 |  | 0.68 | 0.35 – 1.32 | 0.254 |
| **Cardiovascular hospitalization or death^c^** | < 300 | 1.04 | 0.77 – 1.41 | 0.808 |  | 1.13 | 0.85 – 1.50 | 0.414 |
|  | 300 - < 500 | 0.73 | 0.50 – 1.08 | 0.119 |  | 0.67 | 0.45 – 0.99 | 0.042 |
|  | ≥ 500 | 0.71 | 0.47 – 1.08 | 0.110 |  | 0.65 | 0.41 – 1.04 | 0.075 |

RR: rate ratio, 95% CI: 95% confidence interval.

^a^Estimated from weighted Poisson regression models. To examine effect modification by baseline iPTH, baseline iPTH and its interaction with cinacalcet use were added to the weighted Poisson regression models.

In Model 1, to calculate weight, probability of initiating cinacalcet was predicted by age, sex, vintage, primary renal disease, cardiovascular disease, lung disease, liver disease, malignancy, parathyroidectomy, time-varying value of VDRA, phosphate binder, serum Ca, serum inorganic Phosphorus, serum iPTH, dialysate Ca, Kt/V, serum Alb, BMI, Hgb, interaction terms of treatment variables and MBD variables, and visit number. In this model, baseline iPTH was also included in estimating the stabilized weight for probability of initiating cinacalcet.

In Model 2, to calculate weight, probability of initiating cinacalcet was predicted by covariates in Model1 (except for baseline iPTH) plus dementia, other central nervous disease, baseline creatinine and total cholesterol, time-varying values of total iron, ferritin, and CRP, and replaces the composite cardiovascular disease variable with indicators: coronary artery disease, atrial fibrillation, arrhythmia, congestive heart failure, cerebrovascular disease, peripheral vascular disease, aortic disease, pacemaker or other cardiovascular disease. As about 20% were missing in total iron, ferritin, and CRP, the function aRegImpute in R was used to make imputed dataset.[^1^](#_ENREF_1)

^b^Estimated from case-cohort studies

^c^Estimated from cohort study

**References.**

1. Harrell, F. E. I*n Regression modeling strategies: With applications to linear models, logistic regression, and survival analysis*. (Springer-Verlag 2001).

# Table S4. Sensitivity analysis for associations between cinacalcet use and clinical outcomes among patients with baseline iPTH ≥ 300 pg/ml using the marginal structural models^a^

| | Model |  | *Adjusted RR* | *95% CI* | *p-value* | | --- | --- | --- | --- | --- | |  | **Death due to any cause^b^** | 0.75 | 0.55 - 1.03 | 0.073 | | **1** | **Death due to cardiovascular disease^b^** | 0.90 | 0.56 - 1.47 | 0.683 | |  | **Cardiovascular hospitalization or death^c^** | 0.71 | 0.53 - 0.94 | 0.016 | |  | **Death due to any cause^b^** | 0.74 | 0.53 - 1.02 | 0.066 | | **2** | **Death due to cardiovascular disease^b^** | 0.91 | 0.54 - 1.55 | 0.737 | |  | **Cardiovascular hospitalization or death^c^** | 0.71 | 0.53 - 0.94 | 0.016 | |  | **Death due to any cause^b^** | 0.72 | 0.51 - 1.01 | 0.058 | | **3** | **Death due to cardiovascular disease^b^** | 0.94 | 0.56 - 1.57 | 0.815 | |  | **Cardiovascular hospitalization or death^c^** | 0.70 | 0.53 - 0.94 | 0.016 |   RR: rate ratio, 95% CI: 95% confidence interval.  Model 1 includes age, sex, vintage, primary renal disease, cardiovascular disease, lung disease, liver disease, malignancy, parathyroidectomy, time-varying value of VDRA, phosphate binder, serum Ca, serum inorganic Phosphorus, serum iPTH, dialysate Ca, Kt/V, serum Alb, BMI, Hgb, interaction terms of treatment variables and MBD variables, and visit number.  Model 2 includes covariates in Model 1 plus dementia, other central nervous disease, baseline creatinine and total cholesterol and replaces the composite cardiovascular disease variable with indicators: coronary artery disease, atrial fibrillation, arrhythmia, congestive heart failure, cerebrovascular disease, peripheral vascular disease, aortic disease, pacemaker or other cardiovascular disease.  Model 3 includes covariates in Model 2 plus time-varying values of total iron, ferritin, and CRP. As about 20% were missing in total iron, ferritin, and CRP, the function aRegImpute in R was used to make imputed dataset.^1^  ^a^Estimated from weighted Poisson regression models  ^b^Estimated from case-cohort studies  ^c^Estimated from cohort study |
| --- | --- | --- | --- | --- | --- | --- | --- | --- | --- | --- | --- | --- | --- | --- | --- | --- | --- | --- | --- | --- | --- | --- | --- | --- | --- | --- | --- | --- | --- | --- | --- | --- | --- | --- | --- | --- | --- | --- | --- | --- | --- | --- | --- | --- | --- | --- | --- | --- | --- | --- |

**Reference**

1. Harrell, F. E. I*n Regression modeling strategies: With applications to linear models, logistic regression, and survival analysis*. (Springer-Verlag 2001).

# Table S5. Outcomes ascertainment and adjudication

| **Clinical outcomes in the MBD-5D study** |
| --- |
| The clinical outcomes in the MBD-5D study were death due to any cause, death due to cardiovascular disease, and a composite of cardiovascular hospitalization or death due to any cause.^1^ Death due to cardiovascular disease was defined as death due to a cerebrovascular disorder, heart failure, myocardial infarction, sudden death, arrhythmia, aortic disease, or other cardiovascular diseases. Cardiovascular hospitalization was defined as hospitalization due to angina, cerebrovascular disease, heart failure, peripheral vascular disease, arrhythmia, myocardial infarction, silent myocardial ischemia, or other cardiovascular diseases. |
| **Outcomes ascertainment** |
| Potential outcomes were identified primarily through medical records at the participating dialysis facilities. Those records include results of relevant diagnostic and laboratory tests, discharge summaries, and patient referral documents from other healthcare providers. Specific details of clinical outcomes were obtained via a case report form provided to each dialysis facility. Deaths (all participants) and hospitalizations (participants in the subcohort) were reviewed every 6 months. If an outcome was identified then the date and disease that caused the outcome were identified. |
| **Adjudication of Outcomes** |
| Physicians in the dialysis facilities and clinical research coordinators classified clinical outcomes. |

**Reference**

1. Fukuhara, S. *et al.* Mineral and bone disorders outcomes study for Japanese chronic kidney disease stage 5D patients: Rationale and study design. *Ther Apher Dial* **15**, 169-175 (2011).

# Figure S1. Change in the proportion of patients receiving cinacalcet over the study period of 3 years

Visit 0 indicates the baseline (December 2007). The time between visits was 3 months. In January 2008 (within visit 1), cinacalcet was approved for use in clinical practice in Japan. Data were derived from the subcohort (n = 3,276). The numbers of patients analyzed gradually decreased, and it was 2,469 at visit 12, due to death, loss to follow-up, or other reasons.

# Figure S2. Proportion of patients receiving cinacalcet continuously over the study period

The proportion of patients receiving cinacalcet continuously is shown. Three months after the first prescription was the first visit at which patients were considered to be receiving cinacalcet, because the time between visits was 3 months. Data were derived from the subcohort (n = 1,384). The number of patients analyzed gradually decreased, and it was to 252 at the 36th month after the first prescription, due to the end of follow-up, death, loss to follow-up, or other reasons.

# Figure S3. Case-cohort studies and cohort study design

The case-cohort design was used for “death due to any cause” and for “death due to cardiovascular disease” as human resources and financial resources were limited. The case-cohort design allows us as if we analyse data of all the patients (whole cohort, N = 8,229) in terms of statistical efficiency using data of the subcohort patients (the subcohort, N = 3,276) randomly chosen from the whole cohort and data of cases outside the subcohort.

As for “death due to any cause”, 1,226 cases were observed during the study period. Among them, 506 were inside the subcohort and 720 were outside the subcohort. Thus, data from 3,996 patients were analyzed. Similarly, as for “death due to cardiovascular disease”, 462 cases were observed. Among them, 191 were inside the subcohort and 271 were outside. Thus, data from 3,547 patients were analyzed. As for “cardiovascular hospitalization or death due to any cause”, 1,054 cases were observed in the subcohort. CV: cardiovascular.

# Appendix

The following investigators also participated in this study: Nobuo Hashimoto (H•N•MEDIC), Mari Ishida (Kitasaito Hospital), Toshiyuki Date (Date Clinic), Kiyotaka Yabuki (Yabuki Hospital), Hideki Tanida (Tendo Onsen Yabuki Clinic), Fumitoshi Yamauchi (San-ai Hospital), Mikihiko Fujishima (Yahaba Clinic), Tomohito Matsunaga (Eijinkai Hospital), Jun Urae (Ishinomaki Clinic), Hiroshi Kawaguchi (Iwaki Urological Hospital), Ikuo Takahashi (Kisen Hospital), Yoshiko Tanaka (Shinjuku-Koshin Clinic), Hideo Kobayashi (Suda Clinic), Maki Takahashi (Suda Naika Clinic), Tatsuya Nonaka (Seishokai Memorial Hospital), Hideto Emoto (Tokai Hospital), Kyosuke Nishio (Shinkoiwa Clinic), Atsushi Hayama (Moriyama Rehabilitation Hospital), Toshio Shinoda (Kawakita General Hospital Dialysis Center), Takashi Kono (Mihama Narita Clinic), Takahiro Mochizuki (Kameda Medical Center), Yasuo Kimura (Shin-kashiwa Clinic), Noriyoshi Murotani (Chiba Social Insurance Hospital), Satoshi Yamaguchi (Asahi Hospital), Taichi Nakanishi (Kurihama Clinic), Kiyoshi Ozawa (Yokosuka Clinic), Takashi Nagaoka (Sagamihara Clinic), Takao Suga (Bousei Hiratsuka Clinic), Masakazu Suda (Suda Medical Clinic), Yoshikazu Goto (Saiyu Soka Hospital), Michio Kuwahara (Shuwa General Hospital Hemodialysis Clinic), Hiromi Shimoyama (Yuai Clinic), Kimihiko Matsuyama (Misato Kenwa Clinic), Kazue Ueki (Toho Hospital), Kyoko Ito (Heisei Hidaka Clinic), Katsuhiko Miyamoto (Seseragi Hospital), Takashi Ishizu (Tukuba Central Hospital), Shuichi Kikuchi (Ohba Renal Clinic), Masaki Kobayashi (Tokyo Medical University Ibaraki Medical Center), Mitsuyoshi Furuhashi (Maruyama Hospital), Masanori Wakabayashi (Bousei Dai-ichi Clinic), Kazuyoshi Nakamura (Fujidaiichi Clinic), Hirotake Kasuga (Kaikoukai Central Clinic), Itsuo Yokoyama (Nagoya Memorial Foundation Narumi Clinic), Chikao Yamazaki (Masuko Clinic SUBARU), Kijun Nagata (Sawada Hospital), Yasumasa Kawade (Suzuka Kidney Clinic), Toshiaki Kawanaka (Ishikiriseiki Hospital), Yoshihiro Tsujimoto (Inoue Hospital), Mikio Okamura (Ohno Memorial Hospital), Shigeki Okada (Okada Clinic), Senji Okuno (Kidney Center Shirasagi Clinic), Harumi Nagayama (Nagayama Hemodialysis Clinic), Shuji Okazaki (Nagayama Hospital), Yoshinori Tone (Fujii Clinic), Ibuki Yajima (Ibuki Clinic), Kouji Shibuya (Sumiyoshigawa Hospital), Kunihiko Yoshiya (Hara Genitourinary Hospital), Morihiro Kondou (Otowa Kinen Hospital), Satoru Yamazaki (Tojinkai Hospital), Ryoichi Miyazaki (Fujita Memorial Hospital), Katsuhiko Arimoto (Shigei Medical Research Hospital), Misaki Moriishi (Nakajima Tsuchiya Clinic), Takahito Nasu (Tokuyama Central Hospital), Seiichi Obayashi (Kinashi Obayashi Hospital), Yuzuru Sato (Sato Junkankika Naika), Takao Tanaka (Ohji Hospital), Hidetoshi Nakamura (Kokura Daiichi Hospital), Nobuhiko Koga (Shin-Koga Clinic), Harumichi Higashi (St. Mary’s Hospital), Kougi Yuu (Takahashi Naika Clinic), Asako Kitamura (Chikuho Social Insurance Hospital), Tomoji Matsumae (Murakami Memorial Hospital), Katsushige Abe (Jinikai Hospital), Masahiro Kawatomi (Kawatomi Internal Medicine Clinic), Motoko Tanaka (Akebono Clinic), Chisa Nogami (Kumamoto Urological Hospital), Etsuo Yoshidome (Ikeda Hospital), Shinyu Miyagi (Okinawa Daiichi Hospital), Satoshi Nakazato (Chibana Clinic), Yoshiki Shiohira (Tomishiro Central Hospital), and Kiyoyuki Tokuyama (Tokuyama Clinic).
