# Supplementary material for: PTH-dependence of the effectiveness of cinacalcet in hemodialysis patients with secondary hyperparathyroidism
Source: Sci Rep. 2016 Apr 13;6:19612. doi: 10.1038/srep19612 (PMC4829837; doi:10.1038/srep19612)
Supplement: Supplementary Information [file srep19612-s2.doc]

Masashi Suzuki^13^, Yoshindo Kawaguchi^14^, Akira Saito^15^, Yoshiki Nishizawa^16^, Yusuke Tsukamoto^17^, Satoshi Kurihara^18^, Takashi Akiba^19^, Eriko Kinugasa^20^, Yuzo Watanabe^21^, Yoshihiro Tominaga^22^, Takashi Shigematsu^23^, Masaaki Inaba^16^, Jun Minakuchi^24^, Hideki Hirakata^25^, Keitaro Yokoyama^26^, Naoki Kimata^19^, Fumihiko Koiwa^27^, Ryoichi Ando^28^, Junichiro J. Kazama^29^, Takatoshi Kakuta^30^ , Hirotaka Komaba^30^, Daijo Inaguma^22^, Eiji Ishimura^16^, Hideki Tahara^16^, Kazuhiko Tsuruya^31^, and Akira Fujimori^32^.

Division of Nephrology, Shinrakuen Hospital, Niigata, Japan^13^

Division of Internal Medicine, Shiomidai Hospital, Yokohama, Japan^14^

Division of Nephrology, International University of Health and Welfare Atami Hospital, Atami, Japan^15^

Department of Metabolism, Endocrinology, and Molecular Medicine, Osaka City University Graduate School of Medicine^16^

Division of Nephrology, Shuwa General Hospital, Kasukabe, Japan^17^

Division of Nephrology, Tsukinomori Clinic, Saitama, Japan^18^

Department of Blood Purification, Kidney Center, Tokyo Women’s Medical University, Tokyo, Japan^19^

Division of Internal Medicine, Showa University Northern Yokohama Hospital, Yokohama, Japan^20^

Division of Internal Medicine, Kasugai Municipal Hospital, Kasugai, Japan^21^

Division of Transplant surgery and Endocrine surgery, Japan Red Cross Nagoya Daini Hospital, Nagoya, Japan^22^

Division of Nephrology, Department of Internal Medicine, Wakayama Medical University, Wakayama, Japan ^23^

Division of Nephrology, Kawashima Hospital, Tokushima, Japan^24^

Department of Nephrology, Japan Red Cross Fukuoka Hospital, Fukuoka, Japan^25^

Division of Kidney and Hypertension, Department of Internal Medicine, Jikei University School of Medicine, Tokyo, Japan^26^

Division of Nephrology, Showa University Fujigaoka Hospital, Yokohama, Japan^27^

Division of Nephrology, Japan Red Cross Musashino Hospital, Musashino, Japan^28^

Division of Clinical Nephrology and Rheumatology, Niigata University Graduate School of Medical and Dental Sciences, Niigata, Japan ^29^

Division of Nephrology, Endocrinology and Metabolism, Tokai University School of Medicine, Isehara, Japan^30^

Department of Integrated Therapy for Chronic Kidney Disease, Graduate School of Medical Sciences, Kyushu University, Fukuoka, Japan^31^

Blood Purification Center, Konan Hospital, Kobe, Japan^32^
